# Study protocol: Mixed-methods evaluation of a cluster-randomized trial on preventing dysregulated screen use in children under 3

**DOI:** 10.1371/journal.pone.0324012

**Published:** 2025-05-19

**Authors:** Juliane Schemmer, Hanno Krafft, Tobias Maurer, Silke Lange, Anke Emgenbroich, Sean Monks, David Martin, Silke Schwarz

**Affiliations:** 1 Institute for Integrative Medicine, Chair of Medical Theory, Integrative and Anthroposophic Medicine, Department of Human Medicine, Faculty of Health/School of Medicine, Witten/Herdecke University, Herdecke, Germany; 2 Interprofessional Graduate School Integrative Medicine and Health, Health Department, University of Witten/Herdecke, Witten, Germany; 3 BVKJ-Service GmbH, Cologne, Germany; 4 Monks - Ärzte im Netz GmbH, Munich, Germany; 5 University Clinic for Pediatric and Adolescent Medicine, University of Tübingen, Tübingen, Germany; National Research Centre, EGYPT

## Abstract

**Background:**

Screen media use in early childhood is associated with increased risk for health and developmental problems. A preventive intervention during a regular examination in pediatric practices aims to prevent dysregulated screen time by children under three years of age. This protocol describes a process evaluation with the aim of understanding how to implement most effectively a complex intervention to prevent dysregulated screen time in childhood. For this purpose, the process evaluation combines two frameworks to identify both the implementation process and the relevant contextual factors.

**Methods:**

The focus of this process evaluation is a nation-wide, cluster-randomized controlled trial with a complex intervention. Two-thirds of all pediatric practices registered with Professional Association of Pediatricians in Germany received the intervention materials with the option to participate in May 2022, while one-third maintain as usual. Since than an app has been collecting children’s screen time and developmental diagnostic parameters using a longitudinal parent survey. The process evaluation will examine the implementation process following the RE-AIM scheme (Reach, Effectiveness, Adoption, Implementation, and Maintenance) as well as the relevant contextual factors influencing the effectiveness of the implementation process following the Consolidated Framework for Implementation Research. Data collection will include quantitative and qualitative methods. Measurement points are at the start of the intervention, several times during the ongoing intervention and after two years. The two groups of people will be interviewed: Pediatricians who use the intervention in their practice and parents who have received the intervention.

**Discussion:**

This protocol illustrates the process evaluation of a complex, app-based intervention in pediatric practices. It combines the two frameworks RE-AIM and CFIR and thus covers all aspects of the evaluation of the implementation process of a complex intervention.

**Trial register number:**

DRKS00032258; https://drks.de/search/en/trial/DRKS00032258.

## Introduction

Studies show that early screen exposure can lead to serious problems for children’s health and development, but the correlations have not yet been fully explored. Excessive Screen media use in infancy and early childhood is associated with behavioral disorders [1–5], speech development delays [6,7], motor development problems [2,8], obesity [5,9,10], insulin resistance and diabetes mellitus type 2 [11,12], altered cortical EEG activity [13] and sleeping problems [9,14,15]. One study found correlations between screen time according to ScreenQ and the parameters of fractional anisotropy and radial diffusivity in brain white matter tracts related to language, executive function, and emergent reading skills in preschool-aged children [16].

National organizations in Germany, such as the Professional Association of Pediatricians (BVKJ) [17], the Federal Centre for Health Education (BZgA) [18], and the national guideline on screen media use [19] do not recommend screen media use by children under three years of age. They recommend a maximum of 30 minutes per day for three to six year olds. In 2022, screen time for three- to five-year-olds increases, with 93 minutes per day for boys and 83 minutes for girls, according to a survey of mothers of children ages three to 13 years (n = 1,176) [20]. However, the actual screen time exceed the recommendations for infants and toddlers many times over.

Previous studies of intervention to reduce children’s screen time showed inconsistent efficiencies [21]. Educating parents about the dangers of their children’s early screen use can potentially help [22]. However, there is a lack of effective intervention on early prevention. The development of an effective intervention to prevent dysregulated screen time in childhood should consider both the content and context of the intervention [23]. The process evaluation examines these two aspects and will optimize the intervention and provide valuable information for further planning and control.

### The intervention study

The process evaluation refers to the Germany-wide randomized controlled trial (Screen free till 3 abbr. BB3). BB3 is a complex intervention with routine parent education in pediatric practices to prevent active and passive screen media use by children under three years of age. In addition, there is an app-based longitudinal parent survey on screen time and child development. Pediatricians perform this intervention in practices during the regular examination of children aged about six months defined in the child guideline by the Federal Joint Committee of Germany [24]. These examinations check the general state of health and the age-appropriate development of children and adolescents.

First, all practices in the intervention group received a starter pack with the study materials (Return postcard, waiting room poster, 100 signal stickers) in May 2022. The practices participate in the study by self-selection.

- Pediatricians find information about the study and the procedure in the study description. Further information and multiplier training is available on the project website.
- The intervention includes hanging the waiting room poster in the practice and distributing the signal stickers. The pediatricians stick the signal sticker in the children’s examination booklet. The main recommendations of BB3 are to engage the child without the use of screen media at least until the age of three; to enjoy meals free from screen media; to avoid screen media running in the background; and to avoid screen media use themselves in the presence of the child. The signal sticker sticks out like a bookmark and reminds parents of the project at least every time they visit the pediatrician.
- The project website provides scientific information, assistance in implementing the recommendations in the family environment and a parent newsletter with e-mail notification.

The longitudinal evaluation takes place via a parent survey in the app my ‘pediatric practice’ (to German PraxisApp ‘Meine pädiatrische Praxis’) of the BVKJ. The App surveys take place when the child is six months, one year, two years and three years old. The first is pre-interventional. The app survey collects the daily screen times of the parents and the child as well as developmental diagnostic parameters. The developmental diagnostic questions are based on the specifications of examinations [24] and include gross motor skills, fine motor skills, language skills, cognitive skills, and social and emotional skills. In addition, the Short-CIUS [25] is integrated into the app survey as an indicator for an internet-related disorder of the parents. The main study on the effectiveness of the intervention is still ongoing at the time of writing this manuscript.

### Aims and objectives

The process evaluation has the basic aim to investigate the implementation process of the Germany-wide BB3 intervention study in the two dimensions of pediatric practices and parents. The objectives are in detail:

1. Evaluation of the reach, effectiveness, adoption, implementation and maintenance of the BB3 intervention in pediatric practices in Germany.
2. Evaluation of the intervention with regard to the participation of parent-child pairs.
3. Identifying barriers and facilitators to the implementation and development of the BB3 intervention study, including economic, political, social, and individual contextual factors.

## Materials and methods

### Study design

The mixed-methods process evaluation is based on the BB3 intervention and logical model according to the MRC Guidance for process evaluation of complex interventions [26]. The process evaluation, based on the two frameworks RE-AIM [27,28] and Consolidated Framework for Implementation Research (CFIR) [29,30]. It evaluates the implementation process and the relevant contextual factors. The RE-AIM framework assessed the results of the implementation process based on the five criteria: Reach, Effectiveness, Adoption, Implementation, and Maintenance. The CFIR framework evaluates the relevant contextual factors that promote or inhibit the outcomes of the implementation process. This includes the five major domains: Characteristic of Intervention, Outer Setting, Inner Setting, Individual Characteristics, and Implementation Process. For more details see Table 1.

**Table 1:** Framework of the process evaluation of BB3 intervention study.

RE-AIM- and CFIR-Framework	Project definition	Data type and source
**Intervention Characteristics**		
BB3	relative advantage of the intervention compared to standard care, adaptability of intervention	expert interviews, documentary analysis
**Outer Setting**		
external information and strategies	corona pandemic, digitization, media recommendations from BVKJ, regular examinations	literature research, expert interviews, parent interviews
**Inner Setting**		
practices	structural features, networks and communication, climate of implementation	literature research, expert interviews
families	sociodemographic factors	literature research, app survey
**Individual Characteristics**		
pediatricians	personal characteristics	expert interviews
parents	knowledge and conviction, personal characteristics	parent interviews
**Implementation Process**		
Reach	absolute number, proportion and representativeness of participants in the app survey and of the use of the project website	app survey, website analysis
Effectiveness	impact of economic effects in practices, reduction of screen times and associated health problems	expert interviews, app survey
Adoption	absolute number, proportion, and representativeness of participating practices, interested institutions, settings of the persons involved	returned postcards, website analysis, expert interviews
Implementation	applicability of the intervention, fidelity (protocol-compliant implementation in practices), adaptations made to interventions and implementation strategies	telephone survey, documentary analysis, expert interviews, parent interviews
Maintenance	absolute number, proportion and representativeness of participating practices and parents after one year, extent to which the intervention becomes part of the routine organizational practices and guidelines	returned postcards, app survey, expert interviews, parent interviews

BB3: screen free till 3; RE-AIM: Reach, Effectiveness, Adoption, Implementation, Maintenance; CFIR: Consolidated Framework for Implementation Research.

### Recruitment

As part of the main study, two-thirds (2,581) of all pediatric practices registered with BVKJ were randomized to the intervention group. They received the intervention materials in May 2022 and participate in self-selection. All pediatric practices that are not in the intervention group are automatically in the control group and provide treatment as usual. The app recruits parents of children born in 2022 to take part in the app survey. Pediatric practices in Germany are already distributing the app to parents of their patients and using it, e.g., to make appointments. The app registers the parent-child pairs through their pediatrician practice. The previous cluster-randomization assigns the parent-child pairs participating in the parent survey to the intervention and control group. The study recruits pediatricians and parents since May 15, 2022.

### Logic model

**Fig 1:**
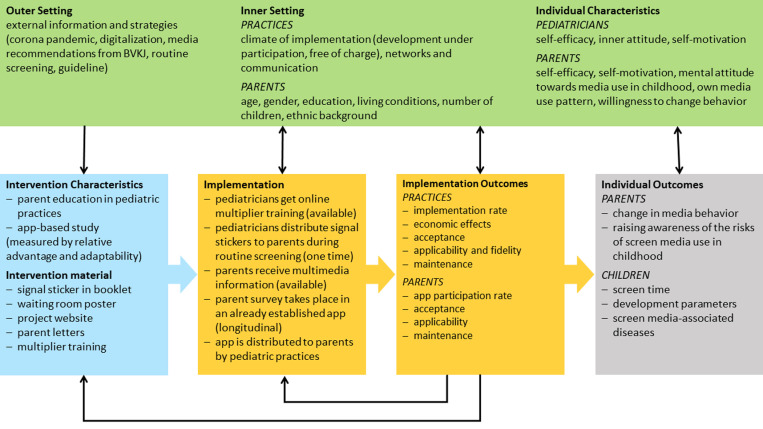
Logic model of BB3 intervention study. The logic model visualizes the BB3 intervention characteristics and material (blue box). It shows the implementation process and outcomes by the RE-AIM analysis (yellow box). The gray box illustrates the mechanisms of impact on the screen use of parents and their children as well as the developmental parameters of the children. The results of the RE-AIM analysis will lead to an adjustment of the BB3 intervention characteristics and material and the implementation process. The economic, political, cultural, social, structural, and individual contextual factors (green box) following the CFIR framework will influence the development of the BB3 intervention characteristics and material, the implementation process and outcomes, and the individual outcomes for parents and children. In addition, the implementation process will influence structures in pediatric practices, and the overall outcomes of the intervention potentially will affect the development of guidelines and the routine organizational practices. BVKJ: Professional Association of Pediatricians; RE-AIM: Reach, Effectiveness, Adoption, Implementation, Maintenance; CFIR: Consolidated Framework for Implementation Research.

The logic model following MRC Guidance [26] visualizes the process of BB3 intervention and underlying contextual factors. Fig 1 shows the implementation process which is evaluated using the RE-AIM analysis. The implementation process will influence individual outcomes for parents and children and therefore the effectiveness of the BB3 intervention. It also will identify the economic, political, cultural, social, structural, and individual contextual factors according to the CFIR framework that will facilitate or hinder the overall intervention process. Table 1 defines the criteria of the RE-AIM analysis and CFIR framework. It also describes the related data collection methods.

### Implementation process

**Fig 2:**
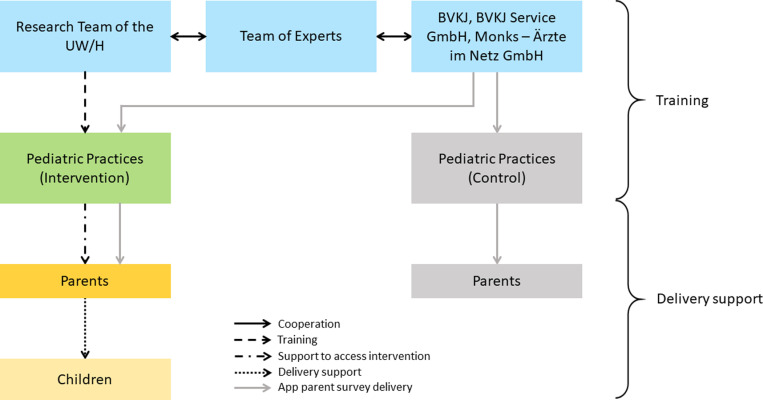
Implementation process of the BB3 intervention study. UW/H: University of Witten/Herdecke; BVKJ: Professional Association of Pediatricians; Monks – Ärzte im Netz (Monks – Doctors on the net): App provider; pediatric practices (intervention): two-thirds of all pediatric practices registered with the BVKJ that have access to the intervention material; pediatric practices (control): one-thirds of all pediatric practices registered with the BVKJ.

A research team at University of Witten/Herdecke (UW/H) has developed the BB3 intervention for the prevention of screen media use by children under 3 years of age. The research team conducted the implementation process in pediatric practices in cooperation with BVKJ Service GmbH and an interdisciplinary team of experts. Fig 2 presents the process of implementing the BB3 intervention. The research team developed the intervention materials and made them available to pediatric practices in the intervention group through mailings. In addition, the research team developed multiplier training for pediatricians and other pediatric practice staff. Since then, the pediatricians have been carrying out the intervention in their practices. This includes a short parent education with advice on the dangers of screen media use in early childhood and giving access to more information. Parents give their children access to intervention by establishing age-appropriate use of screen media. The BVKJ makes the app with the integrated parent survey available to all pediatric practices in Germany. Pediatric practices distribute the app to parents of their patients.

### Data collection and analysis

The BVKJ documentation of the randomized pediatric practices provides the basis for the RE-AIM and CFIR analysis supported by multidimensional quantitative (Table 2) and qualitative methods.

**Table 2:** Quantitative data collection.

Methods	Data source	Data collection
Practice Documentation	return postcard, address list	practice name and practicing physicians; specialty; practice address; use of the app (yes, no); return of the return card to confirm participation in the BB3 intervention (yes, no); reordering of materials; follow-up questions; feedback via contact form on the BB3 website
Telephone survey	call center, survey	position of contact person; receipt of materials; benefit and recommendation of BB3 intervention; need for further help or materials
App Survey	mobile app, parent survey	position of the respondent (mother, father, foster parents, other); child’s living circumstances (child lives permanently with both parents, alternating with both, only with mother or father, with foster parents, other); mother’s and father’s highest education degree (advanced school-leaving certificate, intermediate school-leaving certificate, lower school-leaving certificate, without school-leaving certificate); the duration (hours per day) that respondent spends with the child on working days and weekends; nationality and migration background of the child (yes, no); number and age of siblings; illnesses of family members; age of the child (years and months); week of birth; high-risk pregnancy and hospital treatment after birth (yes, no); congenital diseases of the child; own satisfaction with the child’s development; care by childminder, grandma, aunt or others (yes, no); medical treatment of the child (yes, no); whether the child has its own room (Yes; No, the child sleeps in the parents’ bedroom with them; No, the child shares the room brother/sister/siblings); Short CIUS
Website Analysis	project website	frequency of use; registrations for parent newsletter; contact form on the project website

BB3: screen free till 3; mobile app: PraxisApp “Meine pädiatrische Praxis”, ‘pediatric practice’; BVKJ: Professional Association of Pediatricians; CIUS: Compulsive Internet Use Scale

#### Quantitative data collection.

**Practice documentation and return postcard:** the documentation includes for all intervention practices information about the practice and participation. This data collection started in year 2022.

**Telephone survey:** We conducted a telephone survey via a call center at the beginning of the intervention in July 2022. A random sample of 800 intervention practices participated. The survey included eight questions on the following topics: position of contact person, receipt of materials, implementation, benefit and recommendation of BB3 intervention, need for further help or materials. This data collection has already been completed.

**App survey:** the parent survey takes place via a mobile app since June 2022. The app activates a parent survey at four times depending on the child’s age: 6 months (pre-interventional), 1, 2 and 3 years. The participants answer a questionnaire about their socio-demographics. Paper-pencil survey: As part of a sub-study “BLIKK Media”, we conducted paper-pencil survey on the topic of screen media in pediatric practices with BB3 intervention. The questionnaires from the app survey and the paper-pencil survey are identical.

The integrated short CIUS “Compulsive Internet Use Scale” [25] with five ordinal-scaled questions indicates risky, harmful, or dependent internet use by parents. **Website analysis:** the website analysis evaluates the frequency of use of the project website, registrations for parent newsletter and inquiries via the contact form on the project website.

#### Qualitative data collection.

The qualitative data analysis includes expert interviews [31] and semi-structured parent interviews according to Kruse [32] as well as qualitative feedback from the contact form of the website and professional congresses. The interviews take place face-to-face and by telephone, with the audio sequence recorded for later transcription. The interview language is German. The study recruits pediatricians and parents to participate in the semi-structured interviews from September 1, 2022 to September 1, 2024.

**Expert interviews:** The sample size will be around 15 pediatricians. The group of experts is in itself homogeneous. Hennink and Kaiser (2022) found saturation in a homogeneous sample of 9–17 interviews [33]. We will adjust the sample size according to information power and potential for saturation in the ongoing analysis. We determine the information power based on the criteria according to Malterud et al. (2015): the aim of the study, sample specificity, use of established theory, quality of dialogue, and analysis strategy [34]. The actual sample should be as heterogeneous as possible, which means that participants should have different additional qualifications and years of experience and bring different opinions and ideas to the interview. To determine saturation, we follow the method of Guest et al. (2020) [35].

Pediatricians who themselves run or work in an established pediatric practice are included. Practices should be in the intervention group and thus have received the materials by mail. To find out what motivations pediatricians had to participate or not, we conduct interviews with pediatricians who are actively participating and interviews with pediatricians who are not actively participating. Various methods recruit participants: professional congresses, e-mail and telephone inquiries. An interdisciplinary team develops an interview that contains nine superordinate categories: experience, relevance, acceptability, reach, understandability, applicability and feasibility, facilitation and complications. A separate questionnaire is about the socio-demographics of the participants: level of expertise, gender, age, and years in profession.

**Parent interviews:** semi-structured interviews according to Kruse [32] will be conducted with around 20 parents. Hennink and Kaiser (2022) found saturation in a homogeneous sample of 9–17 interviews [33]. With the sample of parents, the sample is heterogeneous. Parents’ opinions regarding children’s screen media consumption can differ greatly. However, we assume that the interviews with parents will be longer in time and fuller in content than interviews with pediatricians. We will adjust the sample size according to information power and potential for saturation in the ongoing analysis. We determine the information power based on the criteria according to Malterud et al. (2015): the aim of the study, sample specificity, use of established theory, quality of dialogue, and analysis strategy [34]. The actual sample should be as heterogeneous as possible, which means that participants should bring different opinions and ideas to the interview. To determine saturation, we follow the method of Guest et al. (2020) [35].

The sample is composed of parents who have at least one child under the age of three. Parents who have received the signal sticker in their pediatric practice can take part in the interviews. We also conduct interviews with parents who have not yet received the stickers to keep the heterogeneity of the sample as high as possible. The study includes people from different professions, places of residence, ages, nationalities, and ethnic background. Another requirement is that a child resides with the respondent. Various methods recruit participants: project website and public spaces. An interdisciplinary team develops a guideline interview that contains seven superordinate categories: experiences, acceptance, comprehensibility, novelty, feasibility, facilitation and complications. A separate questionnaire is about the socio-demographics of the participants: family function (mother, father, other), gender, age, nationality, number and age of children.

**Website analysis:** people interested in the project can send e-mails to the research team via a contact form on project website. The qualitative analysis will use the feedback on the BB3 intervention.

#### Quantitative data analysis.

The distributions of the collected data will be described separately for the different collectives by means of suitable characteristics of descriptive statistics.

#### Qualitative data analysis.

With the help of qualitative data analysis, we want to find out how effective this form of care is on parents’ media behavior, whether parents accept the recommendations from the intervention and, if necessary, a change of behavior takes place. The qualitative data analysis examined whether parents can implement the recommendations in their everyday life and what makes this easier. In addition, we want to find out what difficulties parents have in keeping their children away from screen media. With the qualitative results, we also want to find out which motivators made parents and pediatricians participate. We investigate how relevant the prevention of screen media use in childhood is from the perspective of pediatricians and what facilitation they experience by using the BB3 materials. The qualitative content analysis is carried out separately from the quantitative analysis. It is intended to complement and contextualize the results of the quantitative analysis, particularly with regard to the contextual factors that influence the effectiveness of the complex intervention.

The interviews are digitally recorded and manually transcribed. All identifiable comments are anonymized in the transcripts. The qualitative content analysis is carried out according to Mayring [36]. The qualitative analysis includes all qualitative data, such as interview transcripts, e-mails, and free text answers from online-surveys. Adjustments and justifications for all changes are noted, analyzed and summarized. The content analysis is based on the questions described in the protocol. The summary is chosen as the basic technique. The analysis follows a flow model with the analysis units: coding unit, context unit and evaluation unit. A system of categories is worked out, based on which the material is coded. The research team interprets the compiled results of the qualitative content analysis with regard to the following questions. The analysis process is checked for compliance with the content analysis quality criteria (reliability, validity).

### Compliance with ethical guidelines

#### Ethics committee.

All described studies and evaluations are performed with the approval of the responsible ethics committee, in accordance with national law, and in accordance with the Declaration of Helsinki of 1975 (in the current, revised version). For the process evaluation, a positive vote (No. 193/2020 of 15.10.2020) of the ethics committee of the University of Witten/Herdecke (Ethik-Kommission der Universität Witten/Herdecke) is available.

#### Data privacy.

Participation in the study is voluntary. All participants will be informed about the study in advance and will give their written consent. The consent to participate in the study can be revoked at any time and without disadvantages. The withdrawal can be made without giving reasons. The UW/H collects the quantitative and qualitative data pseudonymized (app survey) or anonymized (transcribed interviews). The interviews will be transcribed. Any information that could identify the interviewee will be deleted from the transcript. The provisions of confidentiality and the German Federal Data Protection Act (BDSG) are guaranteed.

### Study status

In May 2022, 2,581 pediatric practices registered with the BVKJ received the intervention materials. Since June 2022, participating pediatricians from the intervention group have been placing the signal stickers in the yellow examination booklets at regular examination and parents have been participating in the app survey. The article of the baseline survey of the BB3 intervention study shows that the socio-demographics of the parents-child pairs are similar to the population. In addition, significant correlations were found between high parental screen time and child development parameters [37]. Furthermore, the participation rate of pediatricians and demand for materials is high [38].

## Discussion

To our knowledge, this protocol describes the first process evaluations of an app-based study and complex intervention in outpatient pediatrics. The process evaluation combines two frameworks (RE-AIM and CFIR) and thus covers all aspects of the evaluation of the implementation process of a complex intervention. Implementation is multidimensional: first, structured parent education takes place in pediatric practices, then parents pass the intervention on to their children, which is why the process evaluation examines implementation outcomes and contextual factors at both levels. The quantitative and qualitative data will be collected in a multifaceted manner. The data collection will use methods before, during and two years after the start of the intervention study. The pre-intervention analysis is the baseline survey of parent-child pairs in the app survey. The short-term analysis will determine how well the intervention has started, as well as the participation rate of practices and parents. In addition, it is possible to see at the outset what problems there may have been with implementation in the pediatric practices or with the app survey. The medium-term analysis examines whether there is intervention fidelity and whether the project will be continued. The long-term analysis determines what experience pediatricians and parents have had with the BB3 intervention. The main study examines the screen use and development of children from 6 months to 3 years of age. The study collects participation rates and material orders. It also examined Maintenance until the end of the main study. We conduct the interviews of process evaluation spread over two years.

Interviews will identify extrinsic and intrinsic motivators that entice pediatricians and parents to participate in the BB3 intervention study. The qualitative content analysis should help to understand and explain the outcomes of the quantitative analysis using RE-AIM. The BVKJ is relevant to the profession and should therefore be a strong motivating factor for pediatricians to participate in the BB3 intervention study. Pediatricians exude a natural authority, which may persuade parents to implement the intervention. The materials are designed to provide parents with direct recommendations and support for using screen media and easy access to information. Parents are expected to take the risks seriously, show insight and understanding, and adapt their media behavior and that of their children.

The process evaluation uses RE-AIM and CFIR to determine how well the complex intervention is already implemented in pediatric practices and what actions still need to be taken to fully establish the intervention in outpatient pediatric care. The process evaluation will help understand how to implement a complex intervention in pediatric practices and how to reach parents for participation. The process evaluation will support the sustainability of the BB3 intervention and provide guidance for future complex interventions in pediatric health care research. Due to the novelty of digital research in outpatient pediatrics, this evaluation can be a basis for the development of future studies beyond the boundaries of pediatric health care research. Studies worldwide have shown that high screen media exposure in early childhood is associated with several health problems and developmental delays [2,5,13]. There are guidelines with recommendations on age-appropriate media times to reduce children’s screen time. The World Health Organization guidelines, recommend that children under the age of 2 should not spend any time on screens and children aged 2–4 should spend no more than 60 minutes a day [39]. The American Academy of Pediatrics (AAP) recommends no screen media exposure for children between 0 and 18 months [40]. In Germany, it is recommended to keep children away from screen media in the first 3 years of life [19]. Qualitative research has shown that parents are not well informed about guidelines on screen time for children [41,42]. The BB3 intervention mediates the recommendations of the German guideline. In the interviews, we will ask pediatricians and parents about their attitudes and acceptability towards the no screen till three recommendation. We will ask Pediatricians whether they agree with the recommendation and what they would change. We will evaluate whether parents can implement the recommendation or have difficulties. The process evaluation will examine whether pediatricians and parents find this, in comparison to other guidelines, strong screen limitation appropriate and feasible. The BB3 intervention study could promote the tightening of international WHO or other countries’ recommendations for screen time for infants and young children. This is possible in the case of proven effectiveness in the main study and approval and implementation of the recommendation by pediatricians and parents. In addition, the process evaluation will show how effectively parent education on screen time can be integrated into a routine examination in pediatric practices. Due to the cheap materials and easy implementation, the BB3 intervention will be applicable to other contexts with different healthcare systems. The materials can also be used in other areas, e.g., kindergartens and schools.

Intervention practices received a starter pack of materials containing a postcard by mail. To confirm participation, pediatric practices can stamp the postcard with their practice stamp and send it back to the sender. The number of returned postcards may differ from the actual participation rate, since sending the participation card is another effort. Employees in pediatric practices possibly save time and pediatricians start placing the signal sticker straight away. However, we can approximately determine the participation rate without a return postcard via repeat orders and general e-mail exchanges with the randomized practices.

In July 2022, one month after the start of the study, a telephone survey was conducted with randomized practices, including questions about the arrival of the starter pack, use of the materials and the practice’s initial assessment of the project. Most of the respondents are medical assistants. Especially in large practices, it can happen that a person is reached on the phone who is not involved in the BB3 study in their area of activity and does not know about it. As a result, the results of the telephone survey (participation, recommendation, etc.) may differ from the actual results. The data from the telephone survey serve to provide an overview of the arrival of the materials. Medical assistants accept the starter packs in most cases and pass them on to the pediatricians. In addition, pediatricians are difficult to reach for a survey. Therefore, it is useful to ask this questionnaire about the arrival of materials to medical assistants. We will generate more detailed information in the expert interviews.

A participation rate of parents can be determined via the app survey. This is done by assigning parents to their pediatric practice in the app. In this way, a local allocation of the participating parents can be made via the practice using the practice contact list. In the app survey, the second survey (U6) asks about the signal sticker in the yellow examination booklet. How many parents receive the signal stickers could be determined in real time using QR code tracking, which the QR code on signal stickers and waiting room posters does not yet have. However, conclusions about the number of signal stickers distributed can be drawn approximately from the number of stickers sent to pediatrician practices. An app survey can be a barrier for parents with lower income or limited digital literacy. In addition, not all pediatric practices in Germany use the app and distribute it to parents of patients. Both aspects can lead to a selection bias. To counteract this, we are collecting data as part of a sub-study “BLIKK Media” using a parent questionnaire in paper pencil, with a smaller sample size.
